# The Performance and Fabrication of 3D Variable Cross-Section Channel for Passive Microfluidic Control

**DOI:** 10.3390/mi15081038

**Published:** 2024-08-15

**Authors:** Wenjie Qian, Zhou Zhou, Qing Wang, Wei Shi, Manman Xu, Daoheng Sun

**Affiliations:** 1 School of Mechanical and Automotive Engineering, Anhui Polytechnic University, Wuhu 241000, China; qianwjahpu@163.com (W.Q.); wangqingahpu@163.com (Q.W.); shiweiahpu@163.com (W.S.); 2 Wuhu HIT Robot Industry Research Institute Co., Ltd., Wuhu 241000, China; xumanman@ahpu.edu.cn; 3 School of Electrical Engineering and Automation, Harbin Institute of Technology, Harbin 150001, China; 4 School of Aeronautics and Astronaut, Xiamen University, Xiamen 361102, China; sunhd@xmu.edu.cn

**Keywords:** microfluidic, variable cross-sections channel, finite analysis, 3D printing, diodicity

## Abstract

Passive fluid control has mostly been used for valves, pumps, and mixers in microfluidic systems. The basic principle is to generate localized losses in special channel structures, such as branches, grooves, or spirals. The flow field in two-dimensional space can be easily calculated using the typical Stokes formula, but it is challenging in three-dimensional space. Moreover, the flow field with periodic variable cross-sections channeled of polyhedral units has been neglected in this research field due to previous limitations in manufacturing technology. With the continuous progress of 3D printing technology, the field of microfluidic devices ushered in a new era of manufacturing three-dimensional irregular channels. In this study, we present finite analysis results for a periodic nodular-like channel. The experiments involve variations in the Reynold number (Re), periodic frequency, and comparative analyses with conventional structures. The findings indicate that this variable 3D cross-section structure can readily achieve performance comparable to other passive fluid control methods in valve applications. A 3D model of the periodic tetrahedron channel was fabricated using 3D printing to validate these conclusions. This research has the potential to significantly enhance the performance of passive fluid control units that have long been constrained by manufacturing dimensions.

## 1. Introduction

Due to the significant advantages of the driving methods, such as ease of fabrication, simple operation, long service life, and low cost, passive fluid control techniques have been widely applied in key applications of microfluidic systems, including mixing, separation, and particle capture. Specifically, these applications leverage the local resistance losses caused by the sudden expansion, contraction, or bending of channels to achieve fluid control. The performance of these structures is typically evaluated by comparing the pressure losses in ‘reverse’ (non-promoted) and ‘forward’ (promoted) flow directions at similar volumetric flow rates. This comparison is quantified using a parameter known as diodicity (A.Y. Nobakht, M. Shahsavan, et al., 2013) [1], which measures the efficiency of channels such as the ‘tesla valve’, a one-way valve that allows fluid to flow with lower losses in one direction than in the other. Recently, it has become common to use diodicity for evaluating passive microvalves or pumps, effectively rectifying flow behavior within microfluidic systems. In the Tesla valve study, the researchers used two reflections to study the duality of the valve under different conditions in order to take into account specific factors that may have been overlooked by a single reflection analysis. The researchers concluded that the forward flow can be considered standard flow because there is less loss of flow velocity in the forward flow.

Using topological methods (Gaymann, Montomoli, et al., 2019) [2], it has been demonstrated that diodicity can reach values as high as 3.7 in three-dimensional space; however, there is a lack of fabrication methods to realize this structure on a microscale. Kulkarni, Ranade, et al. (2009) [3] conducted a comprehensive experimental study on the flow and pressure drop characteristics of vortex diodes. They discovered that the diodicity can reach 50 when the port diameters are equal to the chamber height. However, this research was conducted at a micrometer scale with Re (Reynold number) > 10^4^, which exceeds the boundaries of microfluidic systems. Khabarova, Podzerko, et al. (2017) [4] focused on the vortex valve characteristics at low Re < 3000 and observed that the diodicity only reached 1.9. Sweet, Mehta, et al. (2017) [5] presented a prototype of a fully 3D printed check valve featuring a key structure—a membrane driven by local vortices when flow transitions from a large section to a small one. At the millimeter scale, the diodicity can reach 80.6 ± 1.8. Heschel, Mullenborn, et al. (1997) [6] conducted a characterization of diffuser/nozzle structures in silicon and identified them as check valves. They determined that the optimal diffuse angle is 35.3° (crystal orientations of Si), which yields the highest diodicity. Furthermore, both the working direction and diodicity value of this diffuser/nozzle structure are influenced by Re and can be adjusted within the range of 0 to 5. Fadl, Zhang, et al. (2009) [7] utilized the Lattice Boltzmann Equation to compute the diodicity in various channel structures and observed that the nozzle/diffuser configuration effectively rectifies the flow, achieving a maximum diodicity value of 2.1 at Re = 60. However, there is still no universally accepted theory for this nozzle/diffuser structure. Some researchers have suggested that the flow effect in a nozzle/diffuser unit is only achievable in turbulent flow (Koch, Evans, et al., 1998; Gerlach, Schuenemann, et al. 1995; Olsson, Stemme, et al. 1996) [8,9,10], while Singhal, Garimella, et al. (2004) [11] reported that flow rectification could be achieved in a nozzle/diffuser unit for laminar flow. Olsson, Stemme, et al. (1997) [12] suggested that flow rectification in a nozzle/diffuser element is significantly related to the length of the element, while Yang, Chen, et al. (2003) [13] reported that the length of the nozzle/diffuser is irrelevant.

The brief analysis of these fluid fields is typically based on the assumption that the entire channel is parallel and straight. One reason for this phenomenon is the lack of rapid and accurate analytical calculation theory. Additionally, traditional manufacturing methods have not provided solutions for three-dimensional or variable cross-section structures. After years of research, the performance of passive fluid control units has been limited by their low dimension. Recently, 3D printing methods have gradually been employed in microfluidic systems, allowing channels to be designed in almost any shape or size within the equipment’s allowable scope. Using the MJP (multijet print) method, Sochol, Sweet, et al. (2016) [14] integrated a movable membrane into the printed device and achieved flow functionality as a diode. Habhab, Ismail, et al. (2016) [15] designed and fabricated a miniaturized Tesla turbine fluidic pump using the DLP (digital light processing) printing method that could operate at a low Reynolds number of 1000. This development opens up new avenues for researching 3D passive microfluidic structures.

In this paper, we introduce a variable cross-section channel composed of periodic polyhedral units. These polyhedra are generated from parallel nozzles/diffusers extended into three dimensions. We hypothesize that the dimensional effects may lead to an update in diodicity. By adjusting the Reynolds number and applying periodic laws to this nodular channel, our objective is to achieve high diodicity mixing performance within a single channel. Additionally, we consider it a vortex generator and investigate its performance in mixing applications.

## 2. Method and Fabrication

### 2.1. Design of Insert

**Figure 1:**
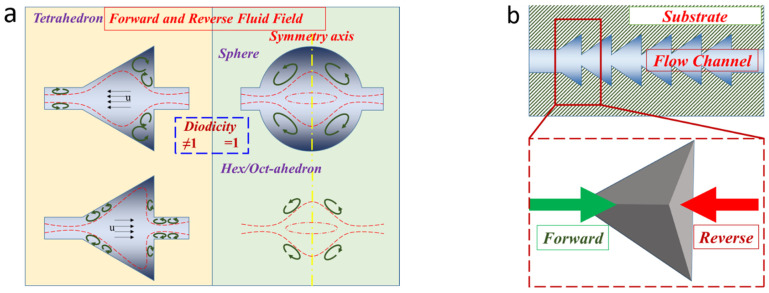
Flow field characteristics utilized in the current study: (**a**) streamline field of symmetric and asymmetric elements; (**b**) cross-sectional view of the variable cross-section channel.

The flow behavior at the microscale is significantly influenced by both inertia and viscous forces, particularly when encountering abrupt changes in the cross-sectional area. When the flow fails to rapidly adjust to sudden changes, vortices are generated, leading to local losses. As depicted in Figure 1a, among the various solid shapes, the tetrahedron with diffuser/nozzle characteristics is the simplest asymmetrical structure that differs from a hexagon or sphere. This asymmetrical characteristic creates significant differences in fluid field between each flow direction. The anisotropic fluid field has an impact on the channel’s diodicity and vortex distribution, which could enhance the performance of mixers and valves. Figure 1b illustrates the structure of the crinoid unit that forms the channel. The typical regular tetrahedron solid unit is selected as the fundamental element.

According to the principles of classical fluid dynamics, the local resistance loss in 2D parallel channels can be elucidated as follows:

$$∆p=\zeta \frac{\rho \mu^{2}}{2}$$

This format is based on the conservation of momentum, where ζ represents the resistance coefficient influenced by the area proportion of the inlet and outlet cross-section, as well as the friction factor and viscous dissipation. Here, *ρ* denotes liquid density, and *μ* signifies flow velocity through the cross-section with a minor area among the inlet and outlet. This demonstrates a sensitive, squared relationship between pressure drop and velocity that is affected by section transformation. When extending this 2D model to 3D space, accurate estimation of energy loss becomes challenging due to significant vortexes and viscous dissipation in 3D edges. To address this issue, the finite element method has been chosen for fluid field analysis in this paper based on Navier–Stokes equations. The software tool we used was Comsol Multiphysics (COMSOL Multiphysics 6.1.lnk, Comsol Inc., Stockholm, Sweden).

**Figure 2:**
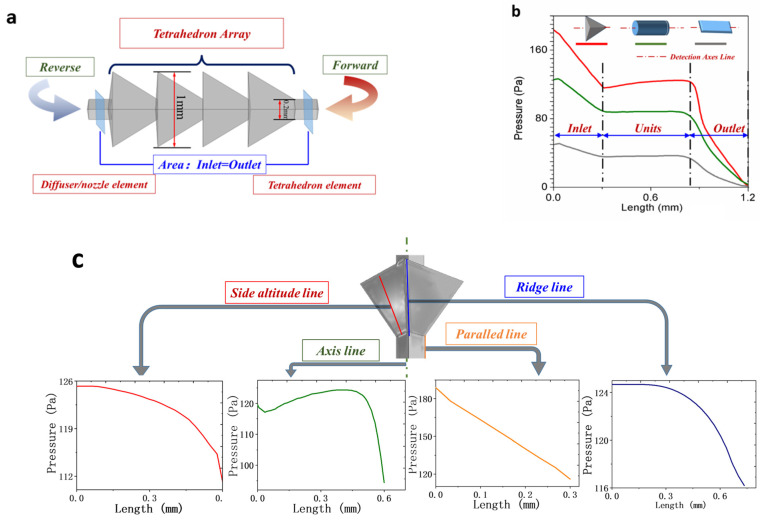
Simulation of pressure drop for typical solid elements: (**a**) the simulation model consists of diffuser/nozzle or tetrahedron elements; (**b**) comparison of pressure drop among tetrahedron, cylinder, and plane structures; (**c**) nonlinear distribution of pressure drop along different edges of a tetrahedron element leading to the formation of local vortices in the flow field.

The model depicted in Figure 2a features a regular tetrahedron with an edge length of 1 mm and a diffuser/nozzle structure as its basic element. To ensure clear contrast, the inlet/outlet of the tetrahedron element is designed with identical structures and scales. The forward direction is defined as the liquid flow from a small section to a large, while the reverse is opposite.

To investigate the flow resistance-induced diodicity or mixing in microchannels, it is essential to compare the 3D variable cross-section channel with conventional ones. Three different channel units—regular tetrahedron, plane rectangular, and circular tube—are evaluated under an interface section feature size of 0.2 mm and a length of 0.6 mm. In this analysis, the inlet flow is standardized to a parallel Reynold number (Re) of 100. We conducted pressure data analysis along the central axis of each channel. As depicted in Figure 2b, a significant pressure drop is observed in the tetrahedron channel, approximately four times greater than that of a plane rectangular channel. This phenomenon occurs predominantly at locations of sudden contraction or expansion in the inlet/outlet channels, where vortices are concentrated. When flowing into the units, each edge of the tetrahedral channel exhibits distinct non-linear pressure characteristics, unlike the linear behavior observed in a straight channel as depicted in Figure 2c. The pressure data from different sources on the tetrahedral edges exhibit unique characteristics rather than following a linear pattern seen at the straight edge. This imbalance in flow gradient leads to vortex formation in this microchannel.

At the local edge of the periodicity channel, a high degree of nonlinearity is observed. Furthermore, the presence of extreme values or switchover points always accompanies nonlinear phenomena. In this study, Di can be calculated using the following equation:

$$Di=\frac{∆P_{r}}{∆P_{f}}=\frac{P_{r.in}-P_{r.out}}{P_{f.in}-P_{f.out}}\;\;\;\;\;\;\;(\mathrm{forward}\;\mathrm{reflection})$$

And

$$Di=\frac{∆P_{f}}{∆P_{r}}=\frac{P_{f.in}-P_{f.out}}{P_{r.in}-P_{r.out}}\;\;\;\;\;\;\;(\mathrm{reverse}\;\mathrm{reflection})$$

where $∆P_{f}$, $∆P_{r}$, $P_{f.in}$, $P_{f.out}$, $P_{r.in}$, and $P_{r.out}$ represent the pressure drop in the forward direction, pressure drop in the reverse direction, average inlet pressure at the forward cross-section, average outlet pressure at the forward cross-section, average inlet pressure at the reverse cross-section, and average outlet pressure at the reverse cross-section, respectively.

### 2.2. Fabrication and Experiment

Conventional soft lithography technology is evidently constrained by dimensional limitations, particularly along the *z*-axis. We have successfully utilized 3D printing to create channels with variable cross-sections, as well as membranes for use in microfluidic pumps and valves. Existing commercial SLA (stereolithography) or DLP (digital light processing) machines are hindered by the inability to remove support structures from microscale channels. To address this requirement, we opted for the MJP (multijet print) method, which relies on a solvent removal process for our structure. The model was designed using Unigraphics NX10.0 (Siemens PLM Software, Plano, TX, USA) and then converted to STL files through its export module. The HD 3510SD industrial printer from 3D Systems (Rock Hill, SC, USA), features a resolution of 375 × 375 × 790 DPI (x-y-z), which meets the accuracy requirements for our project. Visijet Crystal (3D Systems) photopolymer and Visijet S300 wax (3D Systems) were utilized as the structural material and support material. The post-treatment process involves three steps for removing the S300 support wax: (i) dry-heating at 70 °C for 20~30 min; (ii) immersing in 65 °C plant oil with 45 KHz ultrasonic cleaning; (iii) injecting 65 °C hot oil into the channel using a circulating pump to remove residual wax; and (iv) ultrasonically cleaning in soapy water to eliminate any remaining oil.

**Figure 3:**
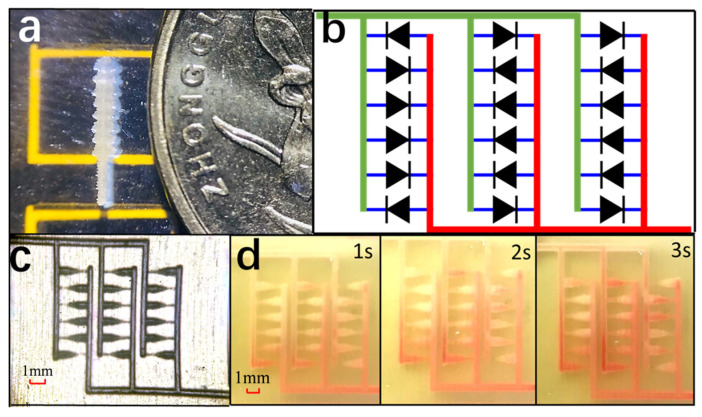
Fabrication and testing of a valve array: (**a**) a 3D-printed nodal solid architecture consisting of eight elements with a cross section of 500 μm; (**b**) a C-type array of 3 × 6 valves (the green and red lines indicate the direction in which the liquid flows in and out, respectively); (**c**) microscopic image of the 3D printed device; (**d**) flow behavior at 30 μL/s, demonstrating selective flow through the valves in the network.

Prior to fabricating the channel structure, we created a shell model with the same shape as the channel. As depicted in Figure 3a, the structural precision of this MJP method has been documented by Kitsakis, Moza, et al. [16], and this shell exhibits a distinct edge of 0.2 mm~1 mm. The integration of multiple elements as components of a functional valve is an indispensable requirement for a passive check valve. To evaluate the capability of 3D printing systems, we developed an integrated diffuser valve array with opposing directions. The microchannel in Figure 3b is equipped with this valve setup, and the 3 × 8 array demonstrates a ‘C’-shaped feature due to the presence of opposing valves. Specifically, Figure 3c shows a microscopic image of a 3D-printed device after post-treatment. The average dimensions of the channels are 200 μm in width and 500 μm in height, while the characteristic diameter of the valves is 500 μm with a diffuse angle of 30°. Dyed DI water was injected into the channel at a rate of 30 μL/s using hematochrome, resulting in an average Reynolds number (Re) for each channel of about 10. In addition, Figure 3d illustrates that during the initial few seconds, liquid flow tends to favor forward-direction valves over reverse ones; furthermore, based on its color intensity level, this array essentially exhibits a ‘C’-shaped model.

To more accurately assess the flow characteristics within the channel, we conducted a series of studies focusing on the variations in flow rate when the diffuser/nozzle node was oriented in both forward and reverse directions under constant pressure conditions. In this research, we employed advanced surface projection micro-stereolithography (PµSL) 3D printing technology (microArch S240) and Visijet crystal materials to meticulously craft a three-dimensional flow channel model with a periodic tetrahedral structure. The model features regular tetrahedral rotation series channels on both sides, with each tetrahedron having an edge length of 1 mm. The two sides are interconnected by a square channel with a side length of 0.25 mm. Additionally, an elastic membrane covers the upper part of the central cylindrical chamber, acting as a pressure chamber.

In our experimental setup, we used two transparent water pipes, each 2 cm long with an inner diameter of 2 mm, connected to either side of the channel. We selected water as the experimental fluid and precisely calculated it using Poiseuille’s law to ensure that the Reynolds number at the channel inlet remained below 30, meeting the low Reynolds number flow conditions of our study. During the experiment, the elastic membrane was fixed on a *Z*-axis pressure platform, undergoing periodic reciprocating motion with a maximum pressure of 0.1 Kpa applied. After every 10 compressions of the elastic membrane, we recorded the volume of liquid passing through the channel to evaluate the characteristics of forward and reverse flows.

**Figure 4:**
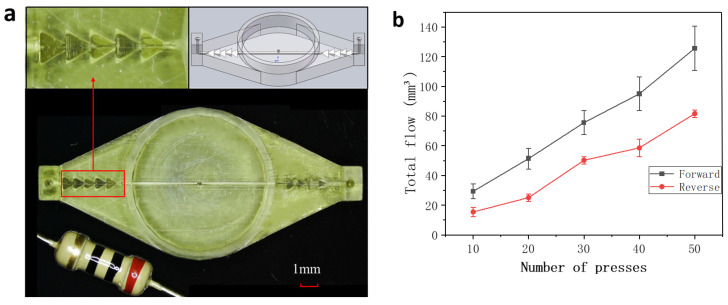
Comparative modeling of positive and negative flow differences: (**a**) 3D printing runner modeling; (**b**) forward and reverse total flow test.

The experimental results show that the 3D-printed channel has an extremely smooth surface and generates significant resistance differences between forward and reverse flows. As shown in Figure 4b, under constant pressure, the laminar flow state in the flow field exhibits good linear stability. The forward flow significantly outperforms the reverse flow, further confirming the potential of our designed channel structure in achieving one-way fluid control.

## 3. Numerical Simulations and Results

**Figure 5:**
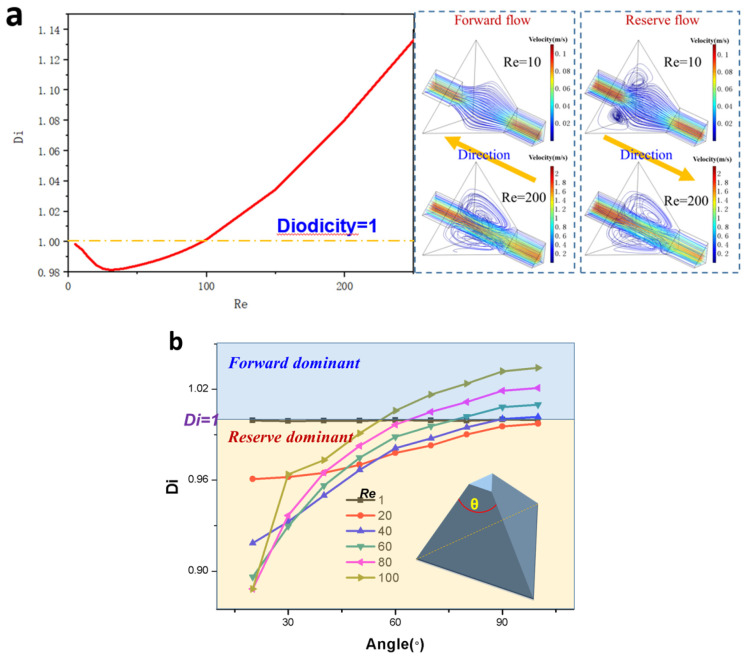
Specificity of one-way flow conduction of a tetrahedron element: (**a**) diodicity curve and the stream line field for Re = 10~200 and angle = 60°; (**b**) diodicity versus Reynolds number and diffuse angle.

By establishing the relationship between Reynolds number and diodicity, the operational range of this unit as a one-way valve was determined in Figure 5a, where the critical Reynolds number is 100, indicating both forward and reverse flow exchange within the valve. Additionally, it illustrates differences in flow streamlines on either side of the critical Reynolds number. It is evident that a vortex always exists in reverse flow but only appears at high Reynolds numbers in forward flow.

In comparison with a parallel channel, the aforementioned experiment on the tetrahedron structure did not yield a high Di at low Re, despite the existence of Di. However, it exhibits similarity to that of a planar dimension. In practical microfluidic system applications, samples typically operate at low Re values below 100, and we can clearly observe a minimum peak in this range with a value less than 1 indicating forward conduction characteristics. Previous research has indicated that in planar dimensions, the Di of diffuser/nozzle structures is influenced by their diffusion angle. However, previous studies have been based on Si crystal lattices, where only angles of 2.6° and 35.3° are considered. This limitation does not exist in 3D printing manufacturing; hence, it is necessary for us to establish the relationship between diffusion angle and Di. As depicted in Figure 5b, the angle range of a tetrahedron can be set from 0° to 120°; however, for practical purposes, the range from 20° to 100° was chosen.

Proposals for forward and reverse flow were tested at various diffuser/nozzle angles and Reynolds number combinations. As the angle (θ) decreases, a pronounced shift towards reverse flow behavior is observed, particularly at higher Reynolds numbers. However, this effect is less significant at ultra-low Reynolds numbers (below 20). For reverse flow conditions, higher values of both θ and Re are preferable. Within the range dominated by reverse flow, specific sets of Reynolds numbers (20, 40, and above 100) should be used for angles greater than 60°, between 30° and 60°, and less than 30°, respectively.

The diffuser/nozzle elements draw inspiration from their application in traditional fluid mechanics, where they effectively regulate fluid flow and facilitate the conversion of pressure and velocity. At the microscale, these elements’ geometric properties are leveraged to enhance the unidirectional flow characteristics of the fluid, known as diodicity. Precise control over the diffuser/nozzle angle is aimed at optimizing fluid flow, resulting in higher diodicity values within the low Reynolds number (Re) range. Specifically, when Re equals 60 and the diffusion angle is set to 60°, a maximum diodicity of 2.1 is observed, highlighting significant advantages of the diffuser/nozzle structure in achieving unidirectional flow control in microfluidic systems.

**Figure 6:**
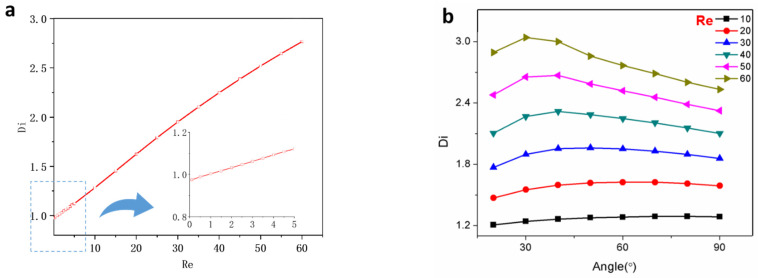
Specificity of one-way flow conduction of the diffuser/nozzle element. (**a**) The diodicity curve and the stream line field in the case of Re = 0.5 to 60 and angle = 60°; (**b**) the diodicity versus Reynolds number and diffusion angle.

Figure 5a illustrates that the microfluidic diodicity is approximately 1 within the range of Re 1 to 100, exhibiting a distinct reverse dominant characteristic. It is well-established that the nozzle/diffuser structure in the plane dimension can effectively rectify the flow, with a maximum diodicity value of 2.1 achieved at Re = 60 when θ is 60°. In Figure 6a, the relationship between diodicity and Reynolds number for the 3D nozzle/diffuser structure is depicted. The findings indicate that flow diodicity becomes prominent at very low Reynolds numbers ranging from 5 to 100. When Re < 1, diodicity almost equals one; however, as Re increases to above 5, Di rapidly escalates until reaching its peak of 2.76 at Re = 60. Unlike the tetrahedron structure, in this case, conduction occurs in the forward direction rather than reverse. Regarding diffuse angle reflection, Figure 6b suggests selecting a relatively smaller angle as Re increases.

**Figure 7:**
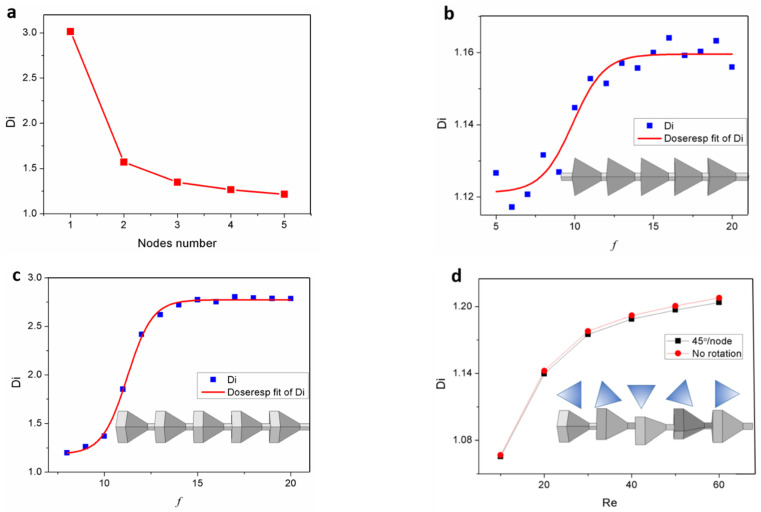
Simulated data on diode characteristics and their influencing factors in an array arrangement: (**a**) the diodicity versus nodes number; (**b**) the diodicity versus *f* in the case of a tetrahedron element; (**c**) the diodicity versus *f* in the case of a diffuser/nozzle element; (**d**) the diodicity versus Re in the case of a rotation diffuser/nozzle element.

The analysis presented in Figure 6 focuses on a single element, necessitating a discussion of its integration performance. To better illustrate the impact pattern of Reynolds number variations on Di, we have chosen five nodes for investigation. Despite one node exhibiting a higher Di value, it was observed to be insufficiently sensitive to changes in Reynolds number. Therefore, by examining these five nodes, we can develop a more comprehensive understanding of the impact pattern of Di resulting from structural Reynolds number variations. Figure 7a shows the performance of multi-element series connected. The result suggested that it is better to use this structure independently than series use. An optimization method was expected to be found for this multi-node combination. Among the numerous controlled parameters, period lengths are an important structural feature of this channel and could be characterized by a frequency, like parameter *f*, which means the node number per 10 mm except both ends. The model is composed of five elements and connected to an equal flow rate source. At Re = 60, the pressure drop property of the tetrahedron node with *f* from 15 to 20 was shown in Figure 7b. Figure 7c shows the results of the periodic diffuser/nozzle channel. The microfluidic diodicity is also related to f. The results suggested that a larger Di could be achieved with the increase of *f*, but the gradient tends to level and can be infinitely approached to the Di that node number = 1. Rotation is another optimization method for the series structure. Figure 7d shows the results of that rotation angle = 45° per node and compared with a parallel structure. Moreover, there is still an enormous gap between a single element and a node combination.

Figure 7a illustrates the performance of a series connection of multiple elements. The results suggest that it is more effective to use this structure independently rather than in series. An optimization method is expected to be developed for this combination of multiple nodes. Among the numerous controlled parameters, the period length is an important structural feature of this channel and can be characterized by a frequency-like parameter *f*, which represents the number of nodes per 10 mm excluding both ends. The model consists of 5 elements connected to a source with equal flow rates. At Re = 60, the pressure drop characteristics of tetrahedron nodes with *f* ranging from 15 to 20 are shown in Figure 7b. Figure 7c presents the results of the periodic diffuser/nozzle channel. The microfluidic diodicity is also related to *f*. The results suggest that a larger Di could be achieved with an increase in *f*, but the gradient tends to level off and can approach infinitely close to Di when the node number equals 1. Rotation represents another optimization method for the series structure; Figure 7d displays the results when each node is rotated at an angle of 45° and compares them with those from a parallel structure. Furthermore, there remains a significant gap between a single element and combinations of nodes.

## 4. Conclusions

This research has successfully utilized 3D printing technology for the design and fabrication of variable cross-section channels in microfluidic systems. Through a combination of experimental and numerical simulations, we have conducted the first analysis of the microfluidic bipolarity exhibited by 3D node components, including tetrahedrons and diffuser/nozzle structures. The experimental results demonstrate that at a Reynolds number (Re) of 60, optimization of the diffusion angle can yield up to 2.76 diodicity (Di), a significant improvement over traditional designs. Furthermore, it has been observed that at low Reynolds numbers (Re < 1), diodicity is nearly equal to one, indicating distinct positive conduction characteristics. As Reynolds number increases, particularly within the range of Re between 5 and 100, diodicity experiences rapid growth, suggesting effective achievement of unidirectional fluid control within this range. In our exploration of multi-node series configuration optimization, we have discovered that individual node performance surpasses that achieved through series usage—a departure from traditional series limit/parallel principles. By adjusting the period length (expressed as the number of nodes per 10 mm or frequency parameter *f*), we have found that greater bipolarity can be attained with increasing *f*; however, as *f* continues to increase beyond a certain point, the rate at which bipolarity increases levels off until it eventually approaches the value obtained from a single node. In conclusion, this study not only showcases the potential application of 3D printing technology in microfluidic system design but also presents novel strategies for enhancing passive fluid control unit performance. Precise manipulation of parameters such as diffusion angle, number of nodes, and rotation angle enables us to achieve highly customized fluid control solutions—potentially offering new design concepts for chemical and biological field practitioners working on two-dimensional systems.

## Data Availability

The original contributions presented in the study are included in the article, further inquiries can be directed to the corresponding author.
